# Whole transcriptome sequencing of normal and tumor bladder tissue samples

**DOI:** 10.1186/gb-2011-12-s1-p23

**Published:** 2011-09-19

**Authors:** Wei Tang, Ludmila Prokunina-Olsson

**Affiliations:** 1 Laboratory of Translational Genomics, Division of Cancer Epidemiology and Genetics, National Cancer Institute, National Institutes of Health, Bethesda, MD 20892, USA

## Background

Bladder cancer is the 9th most common cancer worldwide and the 13th most common cancer-related cause of death. Bladder cancer frequently recurs after the removal of primary carcinomas. This recurrence leads to repeated surgeries and long-term treatment and surveillance, making it the most expensive type of cancer to treat. Genetic factors and environmental factors such as cigarette smoking and occupational exposure to aromatic amines are linked to bladder cancer risk. Genome-wide association studies (GWAS) for bladder cancer have identified multiple genetic variants within genes and regions, including *TP63*, *TERT*-*CLPTMIL* and 8q24.21, to be highly associated with disease risk. Whole transcriptome sequencing (RNA-Seq) is a revolutionary tool for generating a large amount of qualitative and quantitative information, thus helping to explore known and novel transcripts, splicing forms and fusion genes.

## Methods

To understand the genetic and genomic landscape of the GWAS susceptibility regions, we investigated and characterized the entire transcriptome of normal and tumor bladder tissue samples by using powerful massively parallel RNA sequencing. We used an Illumina HiSeq 2000 instrument to sequence six paired samples of normal and tumor bladder tissues. For each of the samples, we generated 50 Gb of 100-bp reads to represent the whole transcriptome.

## Results

Using the Bowtie/TopHat and Samtools packages, we successfully aligned approximately 80% of the total sequence reads against the human genome reference sequence (build 19). Our analysis sought to identify alternative splicing forms, novel exons, non-coding transcripts and chimeric fusion events. Total levels of mRNA in normal and tumor samples were evaluated by Cufflinks analysis based on the Ensembl transcripts database. Multiple splicing isoforms were identified for some of the GWAS susceptibility genes, and some of these isoforms were differentially expressed between the tumor and normal samples. We found that novel transcripts and non-coding RNAs corresponding to gene desert regions such as 8q24 were abundantly expressed. Our next step will focus on validation of these differentially expressed genes and novel transcripts by using quantitative RT-PCR on independent samples.

## Conclusions

Using RNA-Seq, we explored transcripts corresponding to candidate regions identified by bladder cancer GWAS. Some of these transcripts demonstrated splicing variability and differential levels of expression between normal and tumor tissue samples, which might be of importance for bladder cancer.

